# A Differential Reflective Intensity Optical Fiber Angular Displacement Sensor

**DOI:** 10.3390/s16091508

**Published:** 2016-09-16

**Authors:** Binghui Jia, Lei He, Guodong Yan, Yong Feng

**Affiliations:** 1 School of Mechanical Engineering, Nanjing Institute of Technology, Nanjing 211167, China; helei1101@163.com (L.H.); ygd0537@njit.edu.cn (G.Y.); fengyong@njit.edu.cn (Y.F.); 2 Key Laboratory of Road Construction Technology and Equipment, Chang’an University, Xi’an 710064, China

**Keywords:** angular displacement sensor, differential, calibration testing

## Abstract

In this paper, a novel differential reflective intensity optical fiber angular displacement sensor was proposed. This sensor can directly measure the angular and axial linear displacement of a flat surface. The structure of the sensor probe is simple and its basic principle was first analyzed according to the intensity modulation mechanisms. Secondly, in order to trim the dark output voltage to zero, the photoelectric conversion circuit was developed to adjust the signals. Then, the sensor model including the photoelectric conversion circuit has been established, and the influence of design parameters on the sensor output characteristic has been simulated. Finally, the design parameters of the sensor structure were obtained based on the simulation results; and an experimental test system was built for the sensor calibration. Experimental results show that the linear angular range and the sensitivity of the sensor were 74.4 and 0.051 V/°, respectively. Its change rules confirm the operating principle of the sensor well.

## 1. Introduction

Fiber-based devices are widely studied for the advantage in their response to many physical parameters such as displacement, pressure, temperature and electric field [1,2,3]. Recently, intensity-based fiber displacement sensors have received significant attention from the research community for their inherent advantages such as compactness, light weight, small size, non-contact measurement and immunity to a hostile environment [4,5,6,7,8]. Montero [9] demonstrated a radio-frequency self-referencing WDM intensity-based optical sensor operating in reflective configuration. Utilizing intensity modulation mechanisms, Puangmali [10] developed a bent-tip optical fiber and a reflector that can either laterally slide or longitudinally move with reference to the central axis of the fibers. Tosi [11] presented a plastic displacement sensor optical fiber based on the received light intensity after the reflection from the target whose displacement has to be measured. Yasin [12] compared the performance of different types of bundled fiber probes. These theoretical and applied research lead to the improvement of intensity-based fiber displacement sensors. Compared with measuring linear displacement, fewer scholars have focused on intensity-based fiber optic sensors in angular displacement measurement. Khiat [13] and Sakamoto [14] presented a fiber-optic sensor with a micro-lens fixed on the tip of the probe to measure angular displacements, in order to improve the sensitivity and linear range of a fiber optic angular displacement sensor, Sakamoto [15] analyzed the influence of geometrical parameters on the sensitivity and linear range of a fiber optic angular displacement sensor. However, the probe size was enlarged, and how light intensity variation effect on the sensor performances was not taken into account. Guin [16] developed an optical fiber sensor for joint angular measurements. Mingguang [17,18] presented a fiber bundle angular displacement sensor with two parallel multimode fibers placed on both sides of one emitting fiber, although the mothed improved the angular sensitivity without reducing the linear angular range, leaving the axial vibration effects on measurement results out of account, what’s more, the current sensor measurement range still need to improved.

In this paper, we propose a novel differential reflective intensity optical fiber angular displacement sensor. This sensor can directly measure the angular and axial linear displacement of a flat surface. The structure of the sensor probe is introduced, with its basic principle according to the changing intensity. Then the signal conditioning circuit consisting of a light source, a power supply, a photoelectric conversion circuit, a low pass filter was developed to adjust the signals. The sensor model has been established including the photoelectric conversion circuit, and the influence of design parameters on the sensor output characteristic has been simulated. Experimental tests of the static properties of the sensor system were performed, which confirm the operating principle of the sensor.

## 2. Operating Principle of the Sensor

### 2.1. Structure of the Sensor

**Figure 1:**
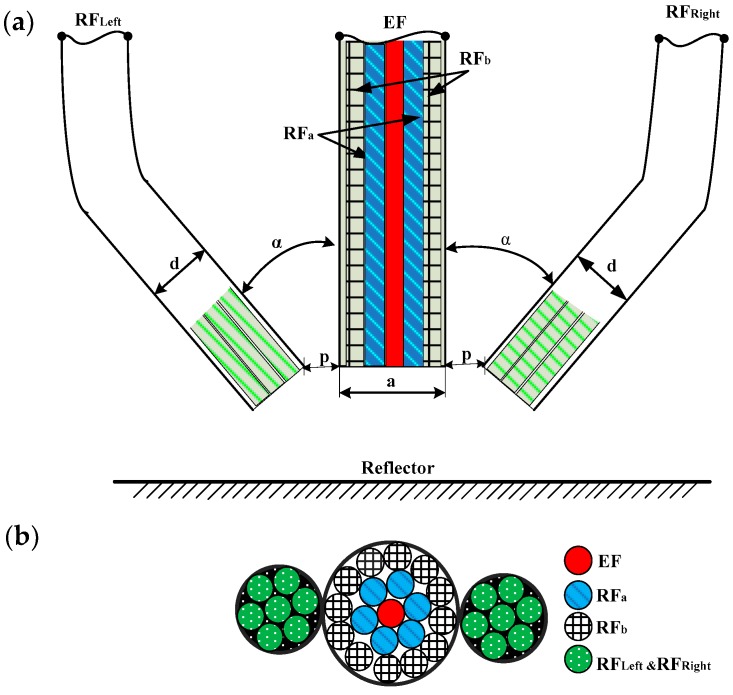
Structure of differential reflective intensity-modulated optical fiber angular displacement sensors: (**a**) sensor architecture; (**b**) the end face of sensor probe.

The structure of the angular displacement sensor probe is shown as Figure 1. It is composed of a double circle coaxial optical fiber bundle perpendicular to the measured plane, a left optical fiber bundle and a right optical fiber bundle; the left optical fiber bundle (RF_Left_) and the right optical fiber bundle (RF_Right_) of an angle *α* to the double circle coaxial optical fiber bundle (the middle bundling is shown in Figure 1b), respectively. The middle fiber bundling was divided into three parts: an emitting fiber (EF), the inner receiving fibers (six fibers, RF_a_) and the outer receiving fibers (twelve fibers, RF_b_). The distance from the fiber end to the reflector can be measured by the division method of the receiving light intensity of RF_a_ and RF_b_.

**Figure 2:**
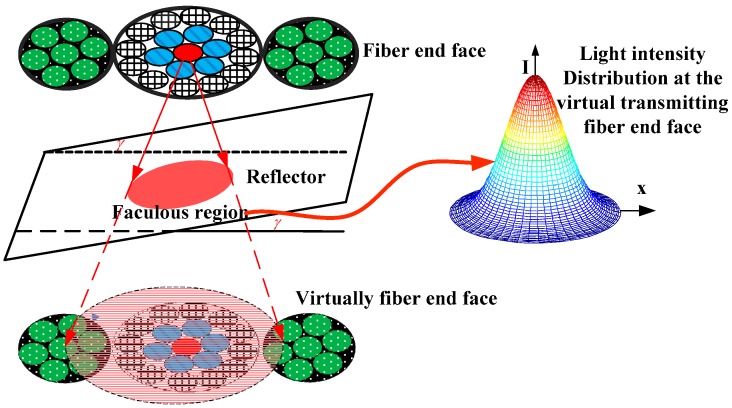
Geometry of the light intensity modulation mechanism.

According to the light intensity distribution of the optical fiber end, the geometric distribution of the emitting fiber emission intensity is shown in Figure 2. Because the middle fiber bundle has a closely packed double circle coaxial arrangement structure, the end faces of RF_a_ and RF_b_ were covered by the reflected light spot, and reflector angular changes have little effect on the axial displacement measurement results [19,20]. The structure introduced in this paper was used to locate the axial displacement of the probe with respect to the reflecting surface. To improve RF_Left_ and RF_Right_ light-receiving efficiency, the RF_Left_ and RF_Right_ fiber bundles were composed of six fibers with same fiber structural parameters.

### 2.2. Differential Reflective Intensity-Modulated of Sensor

**Figure 3:**
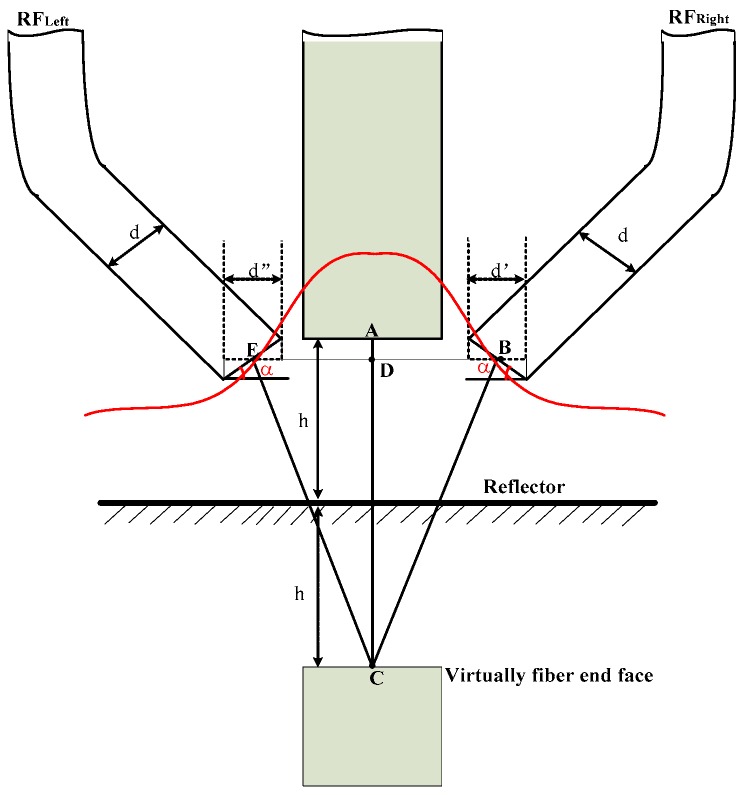
Geometric relationship when reflection plane perpendicular to the transmitting optical.

To simplify the analysis, the radius and numerical aperture of the sensor fibers designed are equal. Considering that the single fiber radius is small, and that the end of the left and right receiving optical fiber bundles are smaller relative to the spot size, it will be treated as a single fiber, and the diameter denoted as d. The diameter of middle fiber bundle is *a*; the spacing of middle fiber bundle and RF_Left_, middle fiber bundle and RF_Right_ were *p* (as shown in Figure 1). The distance from the EF end face to the reflector plane is *h*. Based on the sensor structure shown in Figure 1, and the geometrical relationship of fiber bundles shown in Figure 2 and Figure 3, have $\bar{CB}=\bar{CE}$, $\bar{ED}=\bar{BD}$.

Convert the fiber receiving end face of RF_Left_ and RF_Right_ to effective light receiving surface that is parallel to the reflecting surface, then, the effective receiving surface diameters of RF_Left_ and RF_Right_ are *d*″ = *d*cos(*α*) and *d*′ = *d*cos(*α*), respectively. Note the received light intensity of RF_Left_ and RF_Right_ is *I_Left_* and *I_right_*, as shown in Figure 3, so we have:

(1) $$I_{right}-I_{left}=0$$

*d*′ = *d*cos(*α* − *γ*) (2)

**Figure 4:**
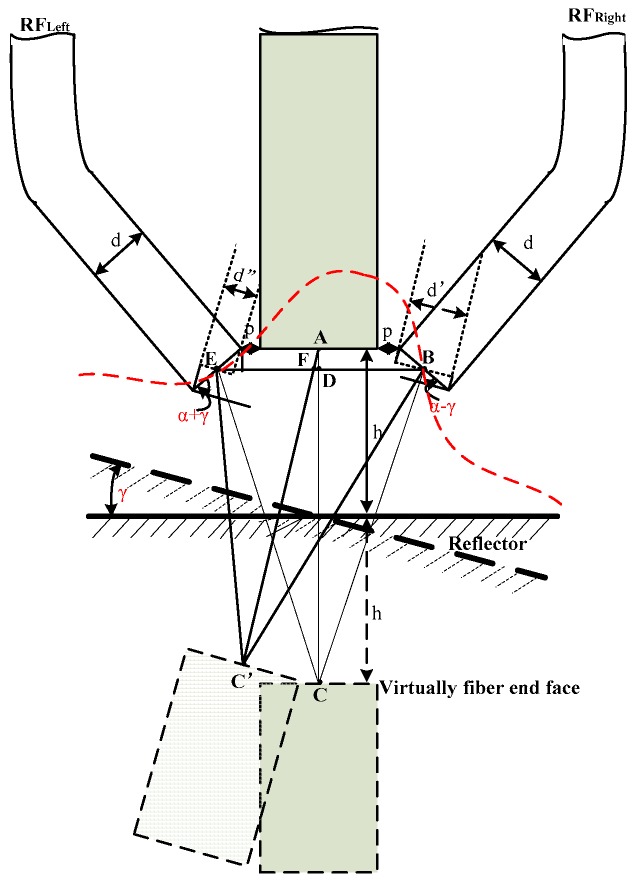
Geometric relationship when the reflection plane has an angle to the transmitting fiber.

The distance between EF and fiber RF_Right_ was gained from the geometrical relationship Δ*C′FB* based on trigonometric functions:

(3) $$\bar{C^{\prime }B}=\sqrt{{\bar{C^{\prime }F}}^{2}+{\bar{FB}}^{2}-2\bar{C^{\prime }F}\cdot \bar{FB}\cdot \cos(\frac{\pi }{2}-\gamma )}$$

where, according to the geometric relationship shown as Figure 4, have:

(4) $$\bar{CD}=\bar{AC}-\bar{AD}=2h-\frac{d}{2\sin(\alpha )}$$

(5) $$\bar{BD}=\frac{a}{2}+p+\frac{d}{2}\cdot \cos(\alpha )$$

(6) $$\bar{AD}=\frac{d}{2}\cdot \sin(\alpha )$$

(7) $$\bar{FD}=\bar{AD}\cdot \sin(\gamma )=\frac{d}{2}\cdot \sin(\alpha )\cdot \sin(\gamma )$$

(8) $$\bar{FB}=\bar{BD}+\bar{FD}=\frac{a}{2}+p+\frac{d}{2}\cdot \left[\cos(\alpha )+\sin(\alpha )\cdot \sin(\gamma )\right]$$

(9) $$\bar{C^{\prime }A}=2h\cdot \cos(\gamma )$$

(10) $$\bar{AF}=\frac{\bar{AD}}{\cos(\gamma )}=\frac{d}{2}\cdot \frac{\sin(\alpha )}{\cos(\gamma )}$$

(11) $$\bar{C^{\prime }F}=\bar{C^{\prime }A}-\bar{AF}=2h\cdot \cos(\gamma )-\frac{d}{2}\cdot \frac{\sin(\alpha )}{\cos(\gamma )}$$

Again, for the RF_Left_ fiber bundle, the diameter of the effective receiving fiber bundle is:

*d*″ = *d*cos(*α + γ*) (12)

The distance between transmitting fiber and RF_Left_ obtained from the geometrical relationship Δ*C′FE* based on trigonometric functions is:

(13) $$\bar{C^{\prime }E}=\sqrt{{\bar{C^{\prime }F}}^{2}+{\bar{EF}}^{2}-2\bar{C^{\prime }F}\cdot \bar{EF}\cdot \cos(\frac{\pi }{2}+\gamma )}$$

where, according to the geometric relations shown in Figure 4 have:

(14) $$\bar{EB}=2\bar{BD}=a+2p+d\cdot \cos(\alpha )$$

(15) $$\begin{array}{c} \bar{EF}=\bar{BD}-\bar{FB}=\frac{a}{2}+p+\frac{d}{2}\cdot \cos(\alpha )-\frac{d}{2}\cdot \sin(\alpha )\cdot \sin(\gamma )\\ =\frac{a}{2}+p+\frac{d}{2}\cdot [\cos(\alpha )-\sin(\alpha )\cdot \sin(\gamma )] \end{array}$$

As many others have shown [21,22,23], the actual optical field end is neither a pure Gauss beam nor a uniform distribution geometric beam, being closer to a blend of the above two beams. For multimode step-index fiber optics, considering above two cases, a universal and practical intensity distribution of the optical field end can be obtained:

(16) $$\phi (r,z)=\frac{K_{0}I_{0}}{\pi \omega (z)}\cdot \exp[-\frac{r^{2}}{\omega {\left(z\right)}^{2}}]$$

where, *φ*(*r,z*) is the luminous flux density of the optical field end that location (*r*, *z*); *K*_0_ is the optical fiber light loss; *I*_0_ is the light strength that light source coupled in the fiber. *ω*(*z*) is the equivalent radius of optical field distribution; and:

(17) $$\omega (z)=\sigma a_{0}\left[1+\xi (\frac{z}{a_{0}})\tan(\theta_{0})\right]$$

where *σ* is the characterization parameter of the optical fiber refractive index profiles. *a*_0_ is the fiber radius; *ξ* is the modulation parameter related to the fiber coupling conditions; *θ*_0_ is the maximum fiber emergence angle.

According to Equation (16), Figure 1 shows the structure of the angular displacement sensor and Figure 4 shows the geometric relationship of the reflection plane, denoting the distance between RF_Right_ and the virtual image of EF $\bar{C^{\prime }B}$ as z_1_, the distance between RF_Left_ and the virtual image of EF $\bar{C^{\prime }E}$ as z_2_; the light receiving area of the two receiving fiber bundles were S_1_ and S_2,_ respectively. Then, the receiving light intensities of RF_Right_ and RF_Left_ are:

(18) $$I_{right}=\rho \iint_{s_{1}}K\phi (r_{1},z_{1})\cdot \exp[-\eta_{1}r_{1}]ds_{1}$$

(19) $$I_{left}=\rho \iint_{s_{2}}K\phi (r_{2},z_{2})\cdot \exp[-\eta_{2}r_{2}]ds_{2}$$

where, $\exp(-\eta_{1}r_{1})$ and $\exp(-\eta_{2}r_{2})$ are the fiber bending light losses. Substituting (16) into (18) and (19) we obtain the receiving light intensity of the left and right receiving fiber:

(20) $$I_{right}=\rho \iint_{s_{1}}K\frac{K_{0}I_{0}}{\pi \omega (z_{1})}\cdot \exp[-\frac{{r_{1}}^{2}}{\omega {\left(z_{1}\right)}^{2}}]\cdot \exp(-\eta_{1}r_{1})ds_{1}$$

(21) $$I_{left}=\rho \iint_{s_{2}}K\frac{K_{0}I_{0}}{\pi \omega (z_{2})}\cdot \exp[-\frac{{r_{2}}^{2}}{\omega {\left(z_{2}\right)}^{2}}]\cdot \exp(-\eta_{2}r_{2})ds_{2}$$

In order to simplify the integral operation of Equations (20) and (21), the light intensity of the fiber center was taken as the average light intensity on fiber surface, then Equations (20) and (21) can be rewritten as:

(22) $$I_{right}=\frac{\rho KK_{0}I_{0}S_{1}}{\pi \omega^{2}(z_{1})}\cdot \exp[-\frac{{r_{1}}^{2}}{\omega^{2}(z_{1})}]\cdot \exp(-\eta_{1}r_{1})$$

(23) $$I_{left}=\frac{\rho KK_{0}I_{0}S_{2}}{\pi \omega^{2}(z_{2})}\cdot \exp[-\frac{{r_{2}}^{2}}{\omega^{2}(z_{2})}]\cdot \exp(-\eta_{2}r_{2})$$

### 2.3. Light Intensity-Voltage Converter

The main function of the photovoltaic module is to convert the light intensity changing signals that caused by the angle variation into a voltage signal. The photovoltaic module designed was used to convert the received reflection light intensity of optical fiber probes that response angular and displacement changes into a voltage signal. 

Burr-Brown (B-B) OPT101 chips were chosen as the photodiode in this system. The OPT101 is a large-area photodiode integrated with an optimized operational amplifier that makes the OPT101 a small, easy-to-use, light-to-voltage device. The photodiode has a very large measurement area that collects a significant amount of light, and thus allows for high-sensitivity measurements. The internal feedback resistor is laser trimmed to 1 MΩ. Using this resistor, the output voltage responsivity, RV, is approximately 0.45 V/μw at 650 nm wavelength. 

The dark errors in the electrical characteristics table include all sources. The dominant source of dark output voltage is the pedestal voltage applied to the noninverting input of the op amp. The OPT101 voltage output is 7.5 mV dc with no light, and increases with increasing illumination. In order to trim the dark output voltage to zero, a low-impedance offset driver (op amp) was used to drive pin 8 (Common) of OTP101 because this node has signal-dependent currents.

**Figure 5:**
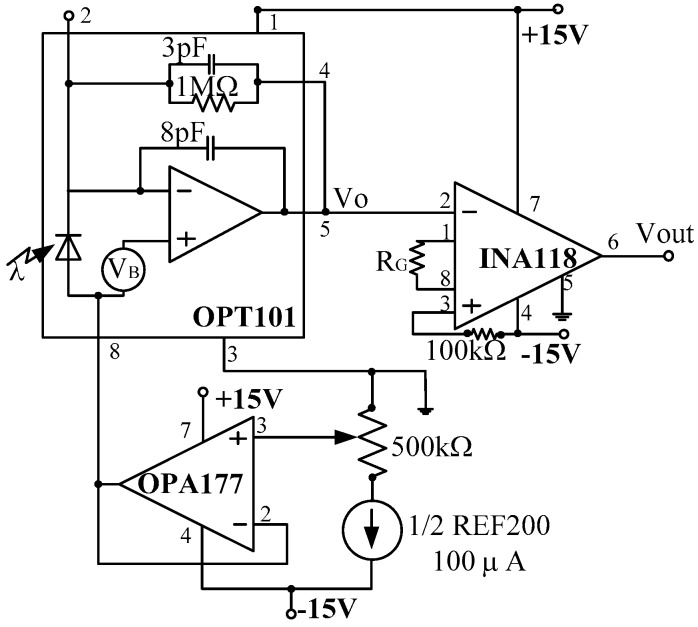
Light intensity to voltage converter circuit connections.

In the photoelectric detection system, the voltage signals were very week after light intensity–voltage conversion, so before the voltage enters data acquisition, it must be fully amplified by suitable amplifying modules. Preamplifier circuit design will directly affect the whole performance of the signal processing circuit. Operational amplifier is mainly used to improve the signal-to-noise ratio of the input signal and to ensure the accuracy of sampling data. Operational amplifier INA118 was choose in this system. The circuit connection is shown in Figure 5, and we have:

(24) $$V_{out}=V_{o}G=V_{o}(1+\frac{50}{R_{G}})=(0.45+\frac{22.5}{R_{G}})I$$

where, *V_o_* = 0.45*I*, *I* is the OPT101 received light intensity, *G* is the magnification times of INA118, *R_G_*is Gain resistance/kΩ.

## 3. Modeling and Simulation of the Sensor

### 3.1. Model of Sensor

Based on above sensor working principle, the output voltage of RF_Right_ and RF_Left_ after light intensity to voltage converter gain:

(25) $$V_{right}=(0.45+\frac{22.5\text{ k}\Omega }{R_{G}})\;I_{right}=(0.45+\frac{22.5\text{ k}\Omega }{R_{G}})\frac{\rho KK_{0}I_{0}S_{1}}{\pi \omega^{2}(z_{1})}\cdot \exp[-\frac{{r_{1}}^{2}}{\omega^{2}(z_{1})}]\cdot \exp(-\eta_{1}r_{1})$$

(26) $$V_{left}=(0.45+\frac{22.5\text{ k}\Omega }{R_{G}})\;I_{left}=(0.45+\frac{22.5\text{ k}\Omega }{R_{G}})\frac{\rho KK_{0}I_{0}S_{2}}{\pi \omega^{2}(z_{2})}\cdot \exp[-\frac{{r_{2}}^{2}}{\omega^{2}(z_{2})}]\cdot \exp(-\eta_{2}r_{2})$$

Synthesizing Equations (25) and (26), to determine the difference voltage of RF_Right_ and RF_Left_:

(27) $$\Delta V=V_{right}-V_{left}=(0.45+\frac{22.5\text{ k}\Omega }{R_{G}})(I_{right}-I_{left})$$

For simplicity, Equations (25)–(27) can be normalized to their maximum value.

### 3.2. Simulation of Optical Fiber Probe Design Parameters Influence on Sensor Measurement Features

Many scholars have studied the transmission characteristics of optical fiber bundles with determined parameters. From Equations (3), (13) and (25)–(27), the characteristics of the sensor are affected by spacing *p* and angle *α* of the receiving optical and the transmitting fiber bundle, the displacement *h* of the transmitting fiber from the reflective surface. In order to obtain the effect law concerning how the above parameters influence the RF_Right_ and RF_Left_ receiving light intensity and the difference of RF_Right_ and RF_Left_, in this paper, without considering the additional bending loss, and noting that *ξ* = 0.5, the numerical aperture of the transmitting fiber NA = 0.37, all the fiber radii of the sensor probes were 150 μm, the normalized characteristic of *V_right_* and *V_left_* was simulated by changing one of the parameters each time.

#### 3.2.1. Effect of *α* on Output Character of RF_Left_ and RF_Right_ Fiber Bundle

**Figure 6:**
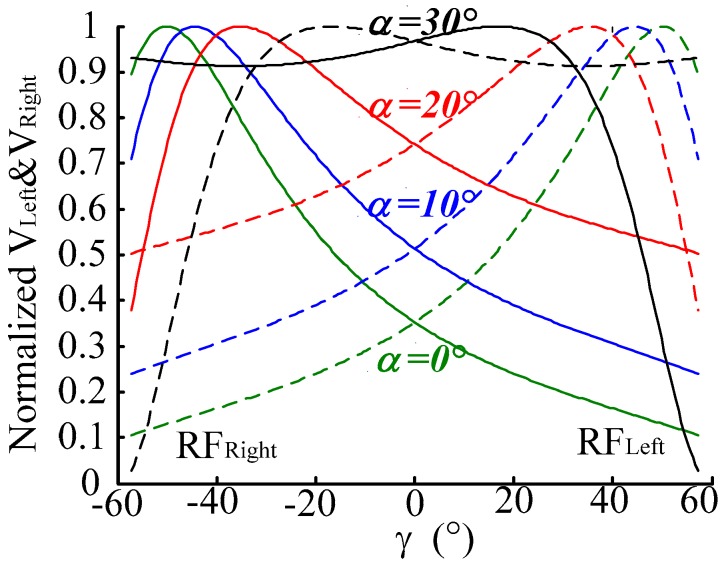
Normalized characteristic curve of *V_Right_* and *V_Left_* when *γ* changes. (*α* is 0°, 10°, 20°, 30° respectively, *p* is 0 μm, *h* is 500 μm).

**Figure 7:**
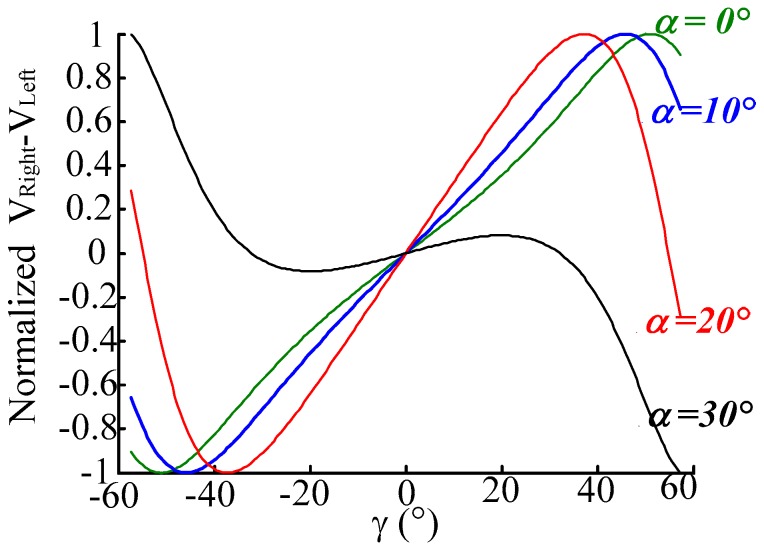
Normalized characteristic curve of *V_Right_* − *V_Left_* when *γ* changes. (α is 0°, 10°, 20°, 30°, respectively, *p* is 0 μm, *h* is 500 μm).

**Figure 8:**
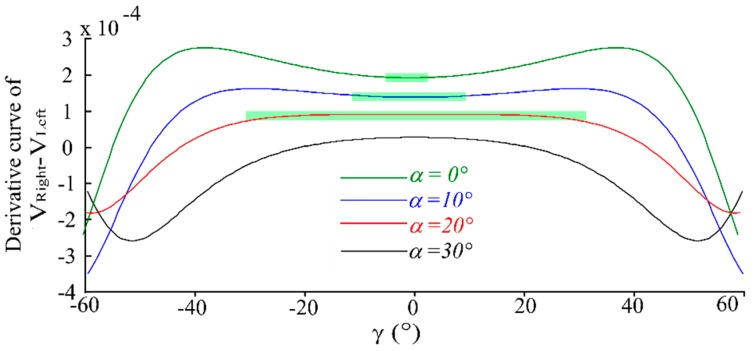
Derivative curve of *V_right_* − *V_Left_*. (*α* is 0°, 10°, 20°, 30°, respectively, *p* is 0 μm, *h* is 500 μm).

Set *α* as 0°, 10°, 20°, 30°, respectively, where *p* is 0 μm and *h* is 500 μm. The normalized characteristic curves of *V_Right_* and *V_Left_* are shown as Figure 6, and the normalized characteristic curve of the intensity difference of RF_Right_ and RF_Left_ is shown in Figure 7. As the simulation results in these figures show, for small *α*, the the receiving light intensities of RF_Right_ and RF_Left_ are more obvious (have higher sensitivity) as the angular displacement is changing, but with large nonlinearity (smaller linear range). This is due to the fact that in a small *α* situation the receiving area of RF_Right_ and RF_Left_ changes quickly when *γ* varies. For a big *α*, as the angular displacement (*γ*) changes the receiving light intensity difference of RF_Right_ and RF_Left_ have higher sensitivity aand better linearity. For the selected fiber parameters in this simulation, when *α* is 20°, the receiving light intensity difference of RF_Right_ and RF_Left_ have higher sensitivity and better linearity (as Figure 7 shows). When *α* is set at 30° the measurement characteristics become worse fast, for this angle beyond the receiving angle of RFs (receiving fibers), which cause the reflected light to not be acceptable by the receiving fibers. It seems that the curves look like they have high sensitivity when *α* = 0° (as Figure 6 shows), but we can see from the derivative curve of *V_Right_* − *V_Left_* in Figure 8, that the sensitivity changes with the different *γ* values when *α* = 0° and *α* = 10°, *α* = 30°, so stability sensitivity is a top priority.

#### 3.2.2. Effect of *p* on the Output Characteristics of the RF_Left_ and RF_Right_ Fiber Bundle

**Figure 9:**
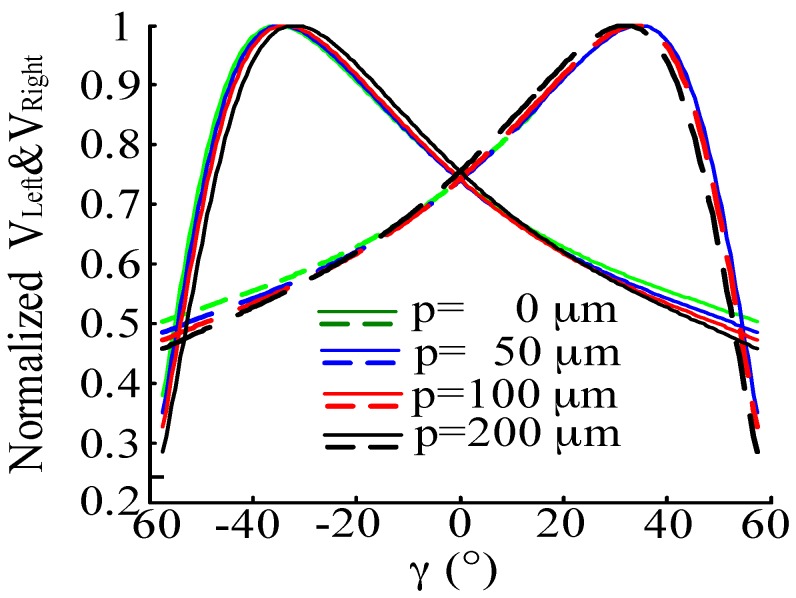
Normalized characteristic curve of *V_Right_* and *V_Left_* when *γ* changes. (*p* is 0 μm, 50 μm, 100 μm and 200 μm, respectively, *α* is 20°, *h* is 500 μm).

**Figure 10:**
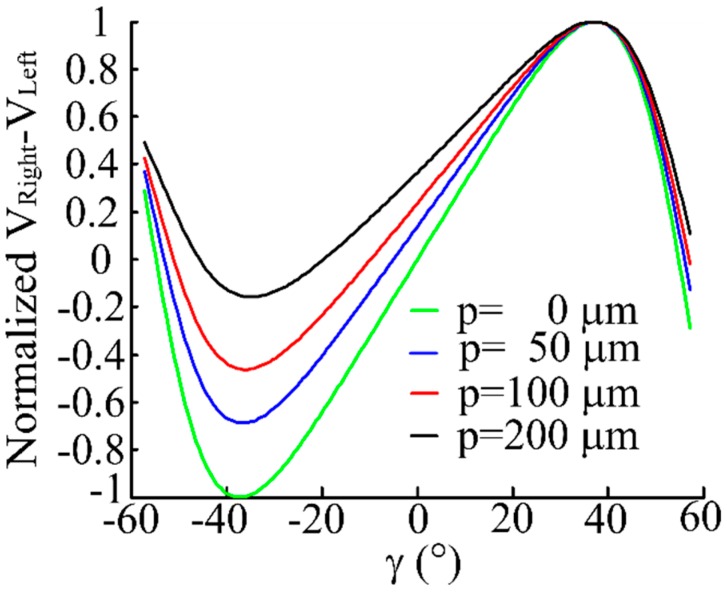
Normalized characteristic curve of *V_Right_* − *V_Left_* when changing *γ*. (*p* is 0 μm, 50 μm, 100 μm and 200 μm, respectively, *α* = 20°, *h* = 500 μm).

Setting parameter *p* at 0 μm, 50 μm, 100 μm and 200 μm respectively, *α* is 20° and *h* is 500 μm. According to Equations (25)–(27), we obtain the normalized characteristic curve of *V_Right_*, *V_Left_* and *V_Right_* – *V_Left_* are shown in Figure 9 and Figure 10. From the simulation results we can see that the receiving light intensity of the RF_Left_ and RF_Right_ fiber bundles are equal when *γ* is 0°, which agrees with the geometric relationships described well in Figure 3 and Figure 4. With RF_Left_ and RF_Right_ keeping away from the EF, the linear range and sensitivity of *V_Right_* − *V_Left_* gets smaller, so a smaller *p* is a top priority.

#### 3.2.3. Effect of *h* on Output Character of RF_Left_ and RF_Right_ Fiber Bundle

**Figure 11:**
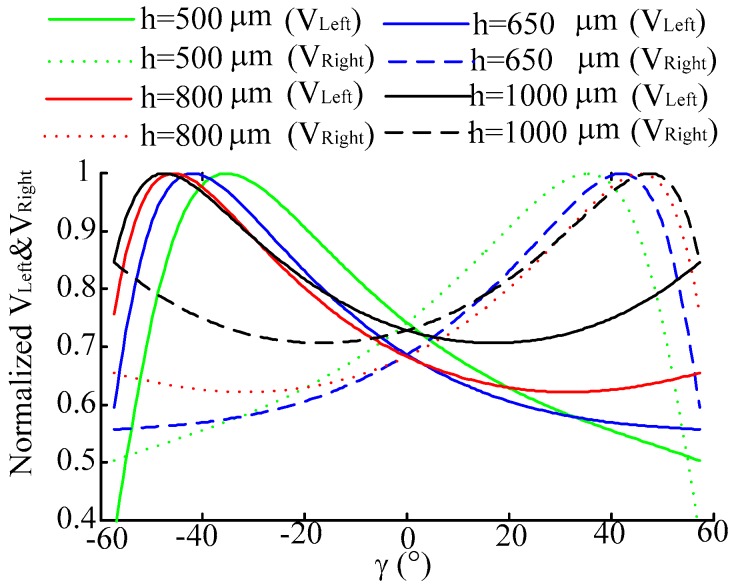
Normalized characteristic curve of *V_Right_* and *V_Left_* when changing *γ*. (*h* is 500 μm, 650 μm, 800 μm, 1000 μm, respectively, and keeping *p* = 0 μm and α = 20° constant).

**Figure 12:**
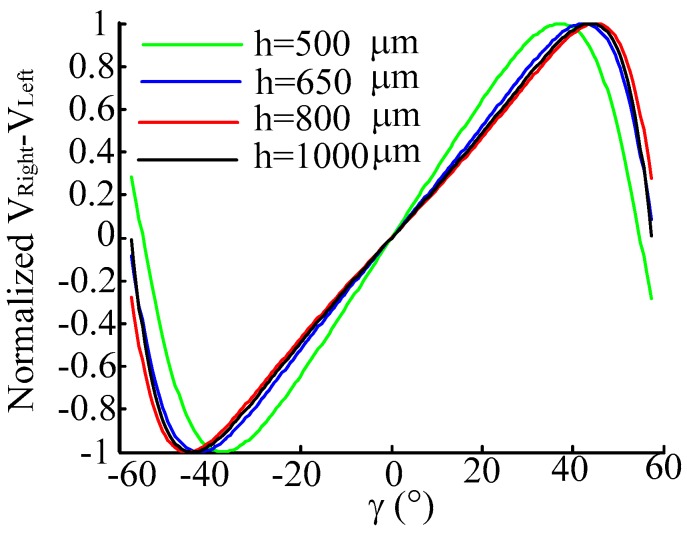
Normalized characteristic curve of *V_Right_* − *V_Left_* when changing *γ*. (*h* is 500 μm, 650 μm, 800 μm, 1000 μm, respectively, and keeping *p* = 0 μm and α = 20° constant).

Set *h* is 500 μm, 650 μm, 800 μm, 1000 μm respectively, keeping *p* = 0 μm and *α* = 20° constant. The normalized characteristic curves of *V_right_*, *V_Left_* and *V_Right_* − *V_Left_* are shown as Figure 11 and Figure 12. From the simulation results we can see that *V_Right_* and *V_Left_* change evidently when *h* is changing, and in the difference of *V_Right_* and *V_Left_* there are also small moves; moreover, *V_Right_, V_Left_* and *V_Right_* − *V_Left_* have a large sensitivity when changing *γ* at small *h*.

According to the above simulation results, for determined design parameters’ of the fiber bundle probe, *V_Right_* and *V_Left_* are affected only by the angle *γ* and the distance *h* from the EF end face to the reflector. The structure parameters of the optical angular displacement sensor are listed in Table 1.

## 4. Experimental Results and Discussion

### 4.1. System Configuration

**Figure 13:**
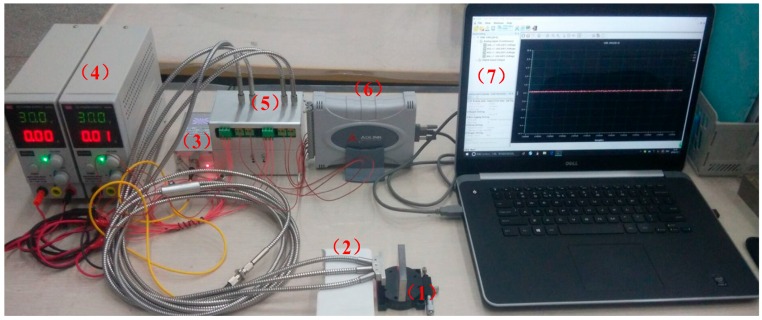
Static measurement experiment system of optical fiber angular displacement sensor.

To verify the performance of the optical angular displacement sensor we have designed, a static measurement experiment system was built as shown as Figure 13. In Figure 13, (1) is the angular displacement change platform; (2) the probe of the sensor; (3) the laser light source (Type: JW3105B; wavelength: 650 nm; power: 10 mW); (4) DC power supply (MCH-305D); (5) light intensity to voltage converter circuit; (6) DAQ module (USB-1901); (7) computer. When the system is working, the 650 nm light irradiates on the angular displacement change platform though EF, then the light is reflected by the plane and received by RF_a_, RF_b_, RF_Left_ and RF_Right_, and then the received light was converted into voltage signals by photoelectric conversion circuit, after that the voltage signal was transmitted to the computer by DAQ module.

**Figure 14:**
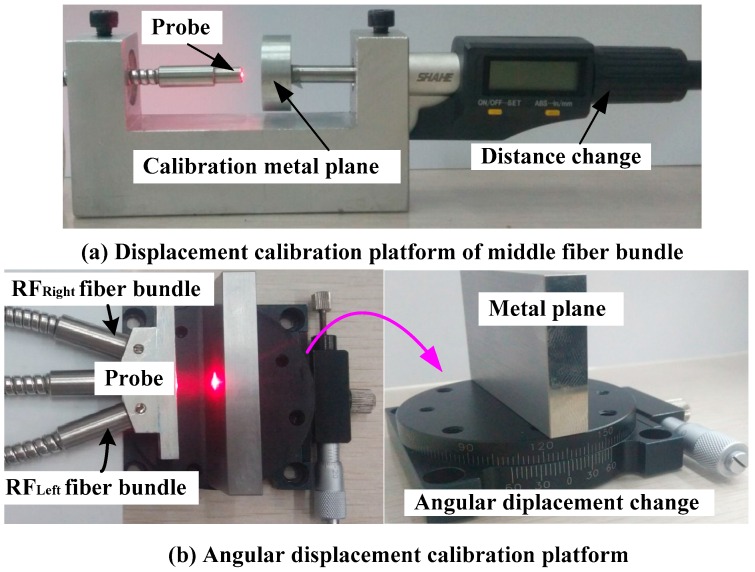
Sensor static calibration station.

In the experiments, the displacement calibration platform was used to calibrate the middle fiber bundle. The platform, shown as Figure 14a, is composed of a digital micrometer (distance change), a calibration metal plane and a metal fixed platform.

The fiber bundle was fixed at one end of the calibration platform by a bolt; the metal plane was taken as the reflector, and is perpendicular to the axis of EF. The distance between the reflector and the end face of EF can be changed by turning the fine adjustment knob of the digital micrometer, and the reading of the digital micrometer is the displacement change of the metal plane. An angular displacement platform shown in Figure 14b was used for sensor static calibration. The platform was composed of a manual goniometer and a metal plane. In the experiments, the sensor fiber bundle was fixed on a plane, keeping the axis perpendicular to the metal plane. The included angle of the metal plane reflector of the EF end face was changed by turning the fine adjustment of the angular displacement change platform (shown in Figure 14b, right), which can be used to measure the displacement of fiber probe end face from the reflector ( the value of the parameter *h*).

### 4.2. The Calibration Experiment Results

#### 4.2.1. Displacement Measurement Calibration Experiment Results

**Figure 15:**
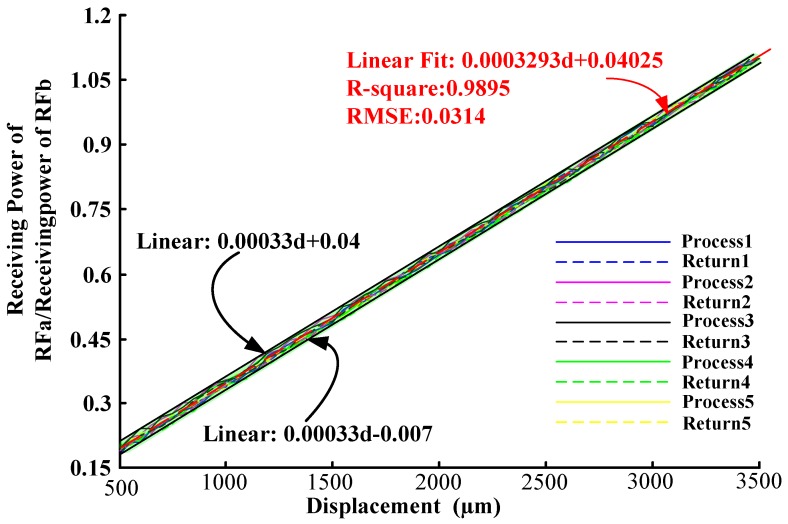
Ratio-displacement curves of the two receiving fibers.

**Figure 16:**
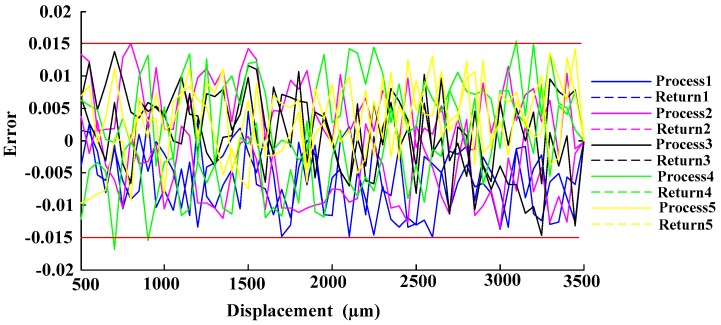
Error of ratio-displacement curve of the two receiving fibers’ deviation from the fitting line.

In the experimental process, the digital screw thread micrometer (with a position accuracy of 0.001 mm) was manipulated driving the reflector plane moving 50 μm in one step in range 0–3.5 mm (experiments were done five times for each process and return, respectively). Then based on the experimental data the process and return ratio-displacement curve of the receiving fiber bundles RF_a_ and RF_b_ are shown in Figure 15. As the figure shows, the sensitivity and linear range are obtained by fitting the experimental RF_a_/RF_b_ to a linear expression with 98% R-square value, the ratio-displacement curve of the receiving fiber bundle RF_a_ and RF_b_ have good linearity in the 0.5–3.5 mm range, and it can be seen from Figure 15 that its sensitivity is 0.31/mm. The error of the deviation of the ratio-displacement curve of two receiving fibers from the fitting line is shown in Figure 16. As shown, the max error of the repeated process and return experiments results is less 0.015, and the hysteresis error is less than 0.5%.

#### 4.2.2. Angular Displacement Measurement Calibration Experiment Results

**Figure 17:**
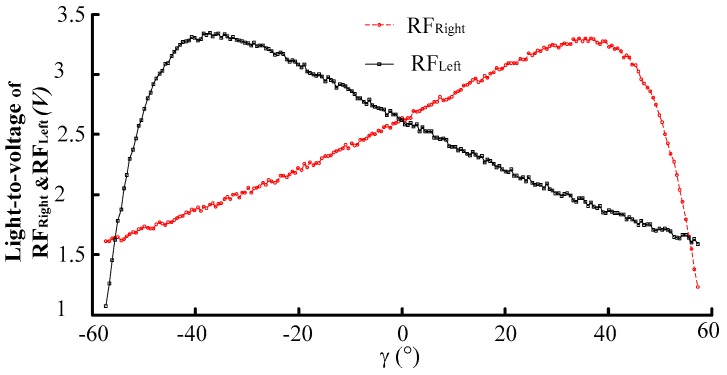
Angular displacement measurement experimental results. (*h* = 500 μm, *p* = 0 μm, *α* = 20°).

**Figure 18:**
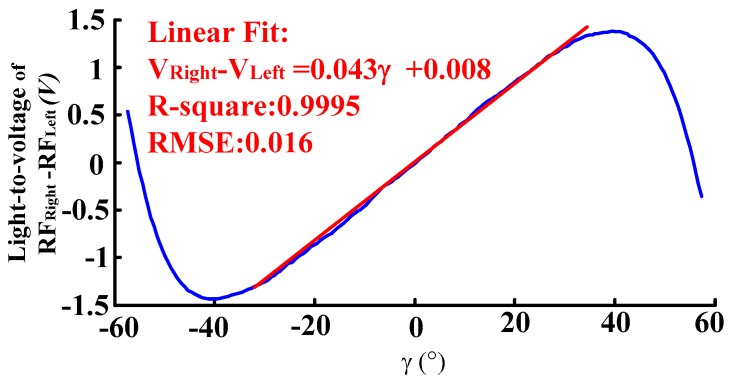
Angular displacement measurement experimental results of *V_right_* − *V_Left_*. (*h* = 500 μm, *p* = 0 μm, *α* = 20°).

**Figure 19:**
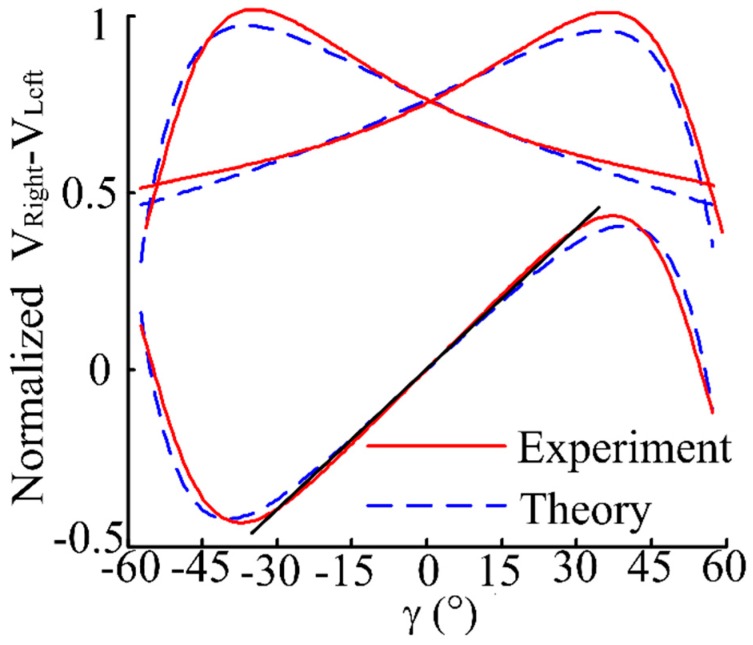
De-noised and normalized calibration result of the angular displacement sensor.

Based on the above displacement measurement calibration experiment results, the fine adjustment of the angular displacement change platform (with a position accuracy of 0.1°) was controlled to change the reflector moving 0.5° in one step over a ±60° range. Then the output voltage curves of RF_Left_ and RF_Right_ receiving light intensity after photoelectric transition were obtained. The output voltage curves of RF_Left_ and RF_Right_ receiving the light intensity after photoelectric transition is shown in Figure 17. The sensitivity and linear range are obtained by fitting the experimental data RF_Right_ − RF_Left_ to a linear expression with 99% R-square value. As can be seen from Figure 18, its linear angular range is 74.4, and its sensitivity is 0.051 V/°. After denoising and normalizing the data of output voltage of RF_Left_ and RF_Right_ and the difference voltage of RF_Left_ and RF_Right_, comparison of experimental results and theoretical results are shown in Figure 19.

It can easily be concluded that the experimental results agree with the theoretical results, which proves the validity of our model. 

## 5. Conclusions

In this paper, we have proposed a novel differential reflective intensity optical fiber angular displacement sensor. The sensor can directly measure the angular and axial linear displacement of a flat surface. The structure of the sensor probe is simple and its basic principle was analyzed according to the intensity modulation mechanism. A photoelectric conversion circuit was developed to adjust the signals. The sensor model has been established including the photoelectric conversion circuit, and how the design parameters affect the sensor output characteristics has been simulated. The experimental tests of the static properties of the sensor system show the measurement range of the sensor is 74.4, and its change rules confirm the operating principle of the sensor well.

## Figures and Tables

**Table 1:** Design parameters of the sensor fiber probe.

Parameters		Value
Emitting fiber (EF)	Fiber radius (nucleus)	150 μm
	Fiber cladding	15 μm
	NA	0.22
Receiving fibers (RFs)	Fiber radius (nucleus)	150 μm
	Fiber cladding	15 μm
	NA	0.37
RF_Left_ and RF_Right_	Diameter of fiber bundle RF_Left_ and RF_Right_	990 μm
	NA	0.37
Fiber type	Standard multimode optical fiber	
Fiber length	1.5 m	
*a*	Diameter of middle fiber bundle	1050 μm
*h*	Displacement to reflector	500 μm
*α*	Degree	20°
